# Optimal sample size planning for the Wilcoxon‐Mann‐Whitney test

**DOI:** 10.1002/sim.7983

**Published:** 2018-10-08

**Authors:** Martin Happ, Arne C. Bathke, Edgar Brunner

**Affiliations:** ^1^ Department of Mathematics University of Salzburg Salzburg Austria; ^2^ Department of Statistics University of Kentucky Lexington Kentucky; ^3^ Department of Medical Statistics University of Göttingen Göttingen Germany

**Keywords:** nonparametric relative effect, nonparametric statistics, optimal design, rank‐based inference, sample size planning, Wilcoxon‐Mann‐Whitney test

## Abstract

There are many different proposed procedures for sample size planning for the Wilcoxon‐Mann‐Whitney test at given type‐I and type‐II error rates α and β, respectively. Most methods assume very specific models or types of data to simplify calculations (eg, ordered categorical or metric data, location shift alternatives, etc). We present a unified approach that covers metric data with and without ties, count data, ordered categorical data, and even dichotomous data. For that, we calculate the unknown theoretical quantities such as the variances under the null and relevant alternative hypothesis by considering the following “synthetic data” approach. We evaluate data whose empirical distribution functions match the theoretical distribution functions involved in the computations of the unknown theoretical quantities. Then, well‐known relations for the ranks of the data are used for the calculations.

In addition to computing the necessary sample size N for a fixed allocation proportion t = n_1_/N, where n_1_ is the sample size in the first group and N = n_1_ + n_2_ is the total sample size, we provide an interval for the optimal allocation rate t, which minimizes the total sample size N. It turns out that, for certain distributions, a balanced design is optimal. We give a characterization of such distributions. Furthermore, we show that the optimal choice of t depends on the ratio of the two variances, which determine the variance of the Wilcoxon‐Mann‐Whitney statistic under the alternative. This is different from an optimal sample size allocation in case of the normal distribution model.

## 1 INTRODUCTION

The comparison of two independent samples is widespread in medicine, the life sciences in general, and other fields of research. Arguably, the most popular method is the unpaired t‐test for two sample comparisons. However, its application is limited. For heavy‐tailed or very skewed distributions, use of the t‐test is not recommended, especially for small sample sizes. For ordered categorical data, comparing averages by means of t‐tests is not appropriate at all. For those situations, a nonparametric test such as the Wilcoxon‐Mann‐Whitney (WMW) test is much preferred.

In order to plan a study for this type of two‐sample comparison, we need to know how many subjects are needed to detect a prespecified effect at least with probability 1 − β, where β denotes the type‐II error probability. If the underlying distributions are normal, a prespecified effect might be formulated as a difference of means. Within a general nonparametric framework, the relative effect (see Section 2) is very often used. However, for a statistics practitioner, it is sometimes difficult to state a relevant effect size to be detected in terms of the nonparametric relative effect. Therefore, we will be using a slightly different approach. Based on prior information F_1_ regarding one group, eg, the standard treatment or the control group, one can derive the distribution F_2_ under a conjectured (relevant) alternative in cooperation with a subject matter expert. This distribution is established in such a way that it features what the subject matter expert would quantify as a relevant effect. In other words, the expert may, but does not necessarily have to, provide a (standardized) difference of means, or a relevant value for the nonparametric relative effect on which the WMW test is based. Or, alternatively, the subject matter expert may simply provide information on a configuration that the expert would consider relevant in terms of providing evidence in favor of the research hypothesis. This information will then be translated into a relevant nonparametric effect. More details on deriving F_2_ based on an interpretable effect to compute the nonparametric effect and the variances involved in the sample size planning are given in Section 4.

For the WMW test, there already exist many sample size formulas. However, most of them require special situations, eg, either continuous data as used in the works of Bürkner et al,^1^ Wang et al,^2^ or Noether,^3^ or they require ordered categorical data as in the works of Fan,^4^ Tang,^5^ Lachin,^6^ Hilton and Mehta,^7^ or Whitehead.^8^ For a review of different methods, we refer to the work of Rahardja et al.^9^ A rather well‐known method for sample size calculation in case of continuous data is given by Noether^3^ who approximated the variance under alternative by the variance under the null hypothesis. A similar approximation was also used by Zhao et al^10^ who generalized Noether's formula to allow for ties. For practical application, however, this approximation may not always be appropriate because the variances under null hypothesis and under alternative can be very different, thus potentially leading to an underpowered or overpowered study. See, eg, the work of Shieh et al^11^ for a comparison of Noether's formula with different alternative methods.

In some other approaches, the sample size is only calculated under the assumption of a proportional odds model for ordered categorical data (eg, the works of Kolassa^12^ or Whitehead^8^), or considering only location shift models for continuous metric data (see, eg, the works of Rosner and Glynn,^13^ Chakraborti et al,^14^ Lesaffre et al,^15^ Hamilton and Collings,^16^ or Collings and Hamilton,^17^ among others). An advantage of our formula (9) in Section 2 for the sample size calculation is its generality and practicality. It can be used for metric data as well as for ordered categorical data, and it even works very well for dichotomous data. Furthermore, our formula does not assume any special model for the alternatives.

Within the published literature, the sample size formulas bearing most similarity to ours are those by Wang et al.^2^ However, their approach is limited to continuous distributions, whereas our approach is based on a unified approach allowing for discrete, as well as continuous data.

A completely different way to approach optimality of WMW tests has been pursued by Matsouaka et al.^18^ They use a weighted sum of multiple WMW tests and determine the optimal weight for each test. Their aim is not an optimal sample size planning including optimization of the ratio of sample sizes, but instead they try to optimally combine a primary endpoint with mortality.

In a two‐sample setting, we sometimes can choose the proportion of subjects in the first group. That is, we can choose t = n_1_/N, where n_1_ is the number of subjects in the first group and N is the total number of subjects. The question that arises is how to choose t in an optimal way. In the work of Bürkner et al,^1^ the optimal t is chosen such that the power of the WMW test is maximized for a given sample size N. On the other hand, in practice, we prefer to choose t in such a way that the total sample size N is minimized for a specified power 1 − β. For the two‐sample t‐test with unequal variances, Dette and O'Brien^19^ showed that the optimal t to maximize the power of the test is approximately

$$t\approx \frac{1}{1+\tau },$$

where τ = σ_1_/σ_0_ is the ratio of standard deviations of the two groups under the hypothesis and under the alternative, respectively. This means that, when applying the t‐test, more subjects should be allocated to the group with the higher variance. Bürkner et al^1^ showed for symmetric continuous distributions under a location shift model that a balanced design is optimal for the WMW test. For general distributions, they observed in simulation studies that, in many situations, the difference between using the optimal t and using a balanced design is negligible.

In most publications, the generation of the alternative from the reference group is not discussed, and instead, the distribution under the alternative is assumed to be known. Here, however, we want to discuss also how we can generate the distribution under the alternative based on the distribution in the reference group and an interpretable relevant effect. In order to motivate the method derived in this paper, let us consider an example with count data, as it appears that most publications on sample size planning focus on ordered categorical or continuous metric data. In Table 1, the data of an advance information F_1_ on a placebo in an epilepsy trial is given where the outcome variable is the number of seizures. We would like to base sample size planning for a new drug on the data X_1,1_,…,X_1,28_ of the advance information F_1_, which comes from a study published by Leppik et al,^20^ as well as Thall and Vail.^21^ For these data, we cannot assume a location shift model, as an absolute reduction of two seizures would be very good for someone with three seizures, but not really helpful for someone with 20 or more seizures. More appropriate would probably be a reduction of the number of seizures by some percentage q, for example q = 50%. Based on this specified relevant effect F_2_(x) = F_1_(x/q), we artificially generate a new data set X_2,1_,…,X_2,28_ whose empirical distribution function ${\hat{F}}_{2}(x)$ is exactly equal to F_2_(x). Basically, the number n_2_ of the artificially generated data is arbitrary (here, n_2_ = 28) as long as ${\hat{F}}_{2}(x)=F_{2}(x)=F_{1}(x/q)$. We will refer to such data as “synthetic” data.

**Table 1:** Number of seizures for 28 subjects from the advance information X_1, k_∼F_1_(x), k = 1,…,28, and for the relevant effect F_2_(x) = F_1_(x/q), where q = 0.5 denotes the percentage of the relevant reduction of seizures to be detected. This means X_2, k_ = [q·X_1, k_]∼F_2_(x), where [u] denotes the largest integer ≤u

Number of counts															
Advance Information
X_1,1_,…,X_1,28_∼F_1_(x)		3,	3,	5,	4,	21,	7,	2,	12,	5,	0,	22,	4,	2,	12
		9,	5,	3,	29,	5,	7,	4,	4,	5,	8,	25,	1,	2,	12
Relevant Alternative
X_2, k_∼F_2_(x) = F_1_(x/q)		1,	1,	2,	2,	10,	3,	1,	6,	2,	0,	11,	2,	1,	6
		4,	2,	1,	14,	2,	3,	2,	2,	2,	4,	12,	0,	1,	6

Most of the methods mentioned before cannot be applied to data such as these as they have been derived under different restrictive assumptions. In particular, methods assuming a location‐shift model cannot be used here. However, application of the method proposed in the present paper does not require specific types of data or a specific alternative because it is based on the observed data and the generated synthetic data, which do not need to follow any particular model. See also the chapter “Keeping Observed Data as a Theoretical Distribution” in the work of Puntanen et al^22^ for a similar approach in the parametric case. More details regarding this data set and the sample size calculation can be found in Section 4.

The rest of this paper is now organized as follows. We first derive a general sample size formula and investigate the behavior of the optimal t. That is, we show in which cases more subjects should be allocated to the first or second group. Then, we apply this method to several data examples with different types of data and provide power simulations to show that, with the sample size calculated by our method, the simulated power is at least 1 − β. Furthermore, we simulate how the chosen type‐I and type‐II error rates affect the value of the optimal allocation rate t.

## 2 SAMPLE SIZE FORMULA

Let X_1i_∼F_1_ and X_2j_∼F_2_, i = 1,…,n_1_, j = 1…,n_2_, be independent random samples obtained on N different subjects, with N = n_1_ + n_2_. The cumulative distribution functions (cdfs) F_1_ and F_2_ are understood as their normalized versions, ie, $F_{i}(x)=\frac{1}{2}(F_{i}^{+}(x)+F_{i}^{-}(x))$, where $F_{i}^{+}$ denotes the right‐continuous cdf and $F_{i}^{-}$ denotes the left‐continuous cdf. By using the normalized version, we can pursue a unified approach for continuous and discrete data; no separate formulas “correcting for ties” are necessary. This unified approach results naturally in the usage of midranks in the formulas for the test statistics; see the works of Ruymgaart,^23^ Akritas et al,^24^ and Akritas and Brunner^25^ for details. We denote by t the proportion of the N subjects that is allocated to the first group. That is, n_1_ = t N and n_2_ = (1 − t)N. Without loss of generality, X_1i_ may be regarded as the reference group and the second group X_2i_ as the (experimental) treatment group. The WMW test is based on the nonparametric relative treatment effect

(1) $$p=\int F_{1}dF_{2}=P(X_{11}<X_{21})+\frac{1}{2}P(X_{11}=X_{21}),$$

which can be estimated in a natural way by its empirical analog $\hat{p}=\int {\hat{F}}_{1}d{\hat{F}}_{2}$. Here, ${\hat{F}}_{i}=\frac{1}{2}({\hat{F}}_{i}^{-}+{\hat{F}}_{i}^{+})$ is the normalized empirical cdf with ${\hat{F}}_{i}^{-}(x)=n_{i}^{-1}\sum_{j=1}^{n_{i}}1_{\left\{X_{i\;j}<x\right\}}$, and ${\hat{F}}_{i}^{+}(x)=n_{i}^{-1}\sum_{j=1}^{n_{i}}1_{\left\{X_{ij}\leq x\right\}}$ the left‐ and right‐continuous empirical cdfs for i = 1,2, respectively. Finally, $1_{\left\{X_{ij}<x\right\}}$ denotes the indicator function of the set {X_i j_ < x}. Using the relation of the so‐called placement $P_{2k}=n_{1}{\hat{F}}_{1}(X_{2k})$ to the overall rank R_2k_ of X_2k_ among all N = n_1_ + n_2_ observations and the internal rank $R_{2k}^{\left(2\right)}$ of X_2k_ only among the n_2_ observations within sample 2, it follows from the asymptotic equivalence theorem (see, eg, theorem 1.3 in the work of Brunner and Puri^26^) that

(2) $$T_{N}=\sqrt{N}(\hat{p}-p)=\sqrt{N}\left[\frac{1}{n_{1}}\left({\bar{R}}_{2\cdot }-\frac{n_{2}+1}{2}\right)-p\right]$$

is asymptotically normal under slight regularity assumptions. Here, ${\bar{R}}_{2\cdot }=\frac{1}{n_{2}}\sum_{k=1}^{n_{2}}R_{2k}$ denotes the mean of the overall ranks R_2k_ in the second sample. For a derivation, we refer, eg, to the works of Brunner and Munzel^27^ or Brunner and Puri^26^ while the placements P_2k_ are considered in more detail at the end of this section in (10). From this theorem, it follows that, asymptotically, the statistic

(3) $$U_{N}=\sqrt{N}\left(n_{2}^{-1}\sum_{j=1}^{n_{2}}F_{1}(X_{2j})-n_{1}^{-1}\sum_{j=1}^{n_{1}}F_{2}(X_{1j})+1-2p\right),$$

which is based on independent random variables, has the same distribution as T_N_. Then, under the null hypothesis H_0_:F_1_ = F_2_, the variance of U_N_ can be written as

(4) $$\sigma_{0}^{2}=\frac{N^{2}}{n_{1}n_{2}}\sigma^{2}=\frac{1}{t(1-t)}\sigma^{2},$$

where $\sigma^{2}=\int F_{1}^{2}dF_{1}-\frac{1}{4}$. This means, T_N_/σ_0_ has asymptotically the same distribution as U_N_/σ_0_, but the distribution of the latter is asymptotically standard normal. To compute the variance of T_N_, in general, we again take advantage of the asymptotically equivalent statistic in (3) and obtain the asymptotic variance

(5) $$\sigma_{N}^{2}=\frac{N}{n_{1}n_{2}}\left(n_{2}\sigma_{1}^{2}+n_{1}\sigma_{2}^{2}\right),$$

where

(6) $$\sigma_{1}^{2}=\mathrm{Var}(F_{2}(X_{11}))=\int F_{2}^{2}dF_{1}-{\left(1-p\right)}^{2},$$

(7) $$\;\sigma_{2}^{2}=\mathrm{Var}(F_{1}(X_{21}))=\int F_{1}^{2}dF_{2}-p^{2}.$$

Clearly, the variance $\sigma_{N}^{2}$ under alternative is a weighted sum of two components, $\sigma_{1}^{2}$ and $\sigma_{2}^{2}$. Both of these components are important for minimizing the sample size, as performed in Section 3, unlike in the parametric case for the t‐test where only the two variances $\sigma_{0}^{2}$ under the null and $\sigma_{1}^{2}$ under the alternative hypotheses are considered.

Based on these considerations, an approximate sample size formula for the WMW test can be obtained similar to the one calculated by Wang et al^2^ for continuous data. Namely, we obtain

(8) $$N=\frac{{\left(\sigma_{0}u_{1-\alpha /2}+\sigma_{N}u_{1-\beta }\right)}^{2}}{{\left(p-\frac{1}{2}\right)}^{2}},$$

where α and β denote the type‐I and type‐II error rates, respectively, and u_1 − α/2_ is the 1 − α/2 quantile of the standard normal distribution.

The quantities p,σ_0_, and σ_N_ in Equation (8) are unknown in general. Moreover, $\sigma_{N}^{2}$ is a linear combination of the two unknown variances $\sigma_{1}^{2}$ and $\sigma_{2}^{2}$ in Equations (6) and (7). To compute these quantities from the distribution F_1_ of the prior information in the reference group and the distribution F_2_ generated by an intuitive and easy to interpret relevant effect, we proceed as follows.

We interpret the distributions of the data as fixed theoretical distributions similar to the parametric case in the works of Seber^28^ ^(p433)^ and Puntanen et al.^22^ ^(pp27‐28^) Therefore, we denote the data from the prior information by $X_{11}^{*},\text{⋯},X_{1n_{1}}^{*}$ and the synthetic data for the treatment group by $X_{21}^{*},\text{⋯},X_{2n_{2}}^{*}$. The corresponding cdfs are denoted by $F_{1}^{*}(x)={\hat{F}}_{1}(x)$ and $F_{2}^{*}(x)={\hat{F}}_{2}(x)$, respectively. Here, ${\hat{F}}_{1}(x)$ denotes the empirical distribution function of the available data $X_{11}^{*},\text{⋯},X_{1n_{1}}^{*}$ in the reference group and ${\hat{F}}_{2}(x)$ the empirical distribution functions of the synthetic data $X_{21}^{*},\text{⋯},X_{2n_{2}}^{*}$ in the treatment group. In this context, “synthetic” means that the data for F_2_ are artificially generated based on the prior information F_1_ and some interpretable relevant effect. We can generate data sets of arbitrary size for F_1_ and F_2_, as long as the relative frequencies or probabilities remain unchanged. Because we assume that our synthetic data represent fixed distributions and not a sample, we can calculate the variances $\sigma_{1}^{2}$, $\sigma_{2}^{2}$, and σ ^2^, as well as the relative effect p exactly. To emphasize that these quantities are not estimators but rather the true parameters based on the synthetic data, we will denote these quantities by $\sigma^{2*},\sigma_{1}^{2*},\sigma_{2}^{2*}$, and p ^∗^.

By using the relations N t = n_1_ and N(1 − t) = n_2_, the sample size formula from Equation (8) is then rewritten as

(9) $$N=\frac{{\left(\sigma^{*}u_{1-\alpha /2}+u_{1-\beta }\sqrt{t\sigma_{2}^{2*}+(1-t)\sigma_{1}^{2*}}\right)}^{2}}{t(1-t){\left(p^{*}-\frac{1}{2}\right)}^{2}}.$$

The variances and the relative effect can be easily calculated by using a simple relation between ranks and the so‐called placements $P_{1k}=n_{2}{\hat{F}}_{2}(X_{1k})$ and $P_{2k}=n_{1}{\hat{F}}_{1}(X_{2k})$, which were introduced by Orban and Wolfe.^29^, ^30^ The placements were first defined only for continuous distributions, but were later generalized to include discrete distributions. For details, see, eg, the work of Brunner and Munzel.^27^ To this end, let $R_{ik}^{*}$ denote the overall rank of $X_{ik}^{*}$ among all n_1_ + n_2_ = N synthetic data, and $R_{ik}^{*(i)}$ the ranks within the ith group, i = 1,2. Furthermore, let ${\bar{R}}_{i\cdot }^{\;*}=\frac{1}{n_{i}}\sum_{k=1}^{n_{i}}R_{ik}^{*}$, i = 1,2, denote the rank means. Then, the placements $P_{ik}^{*}$ can be represented by these ranks as

(10) $$P_{ik}^{*}=R_{ik}^{*}-R_{ik}^{*(i)},$$

i = 1,2;k = 1,…,n_i_. Finally, by letting $F_{i}^{*}(x)={\hat{F}}_{i}(x)$, the quantities in the sample size formula (9) can be calculated directly as follows:

(11) $$\;p^{*}=\int F_{1}^{*}dF_{2}^{*}=\frac{1}{N}\left({\bar{R}}_{2\cdot }^{\;*}-{\bar{R}}_{1\cdot }^{\;*}\right)+\frac{1}{2},$$

(12) $$\sigma^{2*}=\int {\left(F^{*}\right)}^{2}dF^{*}-\frac{1}{4}=\frac{1}{N^{3}}\sum_{i=1}^{2}\sum_{k=1}^{n_{i}}{\left(R_{ik}^{*}-\frac{N+1}{2}\right)}^{2},$$

(13) $$\;\sigma_{1}^{2*}=\int {\left(F_{2}^{*}\right)}^{2}dF_{1}^{*}-{\left(1-p^{*}\right)}^{2}=\frac{1}{n_{1}n_{2}^{2}}\sum_{k=1}^{n_{1}}{\left(P_{1k}^{*}-{\bar{P}}_{1\cdot }^{\;*}\right)}^{2},$$

(14) $$\;\sigma_{2}^{2*}=\int {\left(F_{1}^{*}\right)}^{2}dF_{2}^{*}-{\left(p^{*}\right)}^{2}=\frac{1}{n_{1}^{2}n_{2}}\sum_{k=1}^{n_{2}}{\left(P_{2k}^{*}-{\bar{P}}_{2\cdot }^{\;*}\right)}^{2}.$$

The cdf F ^∗^ is the distribution function of the combined synthetic data from both groups. Note that, for computing the variances, we do not divide by N − 1 or n_i_ − 1, but rather by N or n_i_, i = 1,2 because the distributions of the synthetic data are considered as fixed theoretical distributions similar to the parametric case in the work of Puntanen et al.^22^(pp27‐28)

## 3 MINIMIZING N

### 3.1 Interval for the optimal design

In Section 2, we have derived a formula for the sample size N given type‐I and type‐II error rates α and β, respectively. In practice, we sometimes have the opportunity to choose how many subjects should be allocated to the first group and how many to the second. The question in such a situation is how the proportion t = n_1_/N should be chosen to minimize N. Bürkner et al^1^ aimed at finding the optimal t such that the power is maximized for a given sample size N. Although both questions lead to essentially the same answer, we prefer to minimize the sample size as this question arises more naturally in sample size planning.

Technically, an exact solution to this problem is possible, but it is not feasible to write down the solution in closed form anymore, and it does not give us much information about the behavior of the solution. However, it is possible to provide an interpretable interval for the optimal allocation rate $t_{0}=\arg\min_{t\in (0,1)}\;N(t)$. For that, we only have to assume that the power 1 − β is greater than 50% and we distinguish between the cases σ_1_ = σ_2_ and σ_1_ ≠ σ_2_. Note that the variances $\sigma_{1}^{2}$ and $\sigma_{2}^{2}$ can be quite different even if the variances of F_1_ and F_2_ are the same. If we allow unequal variances for F_1_ and F_2_, it is even possible that $\sigma_{1}^{2}=0$ and $\sigma_{2}^{2}=1/4$ occurs where 1/4 is the largest possible value for the variances $\sigma_{i}^{2}$, i = 1,2.

The assumption on the minimal power could be weakened to assuming that the numerator of N(t) is not zero. One then only needs to distinguish the cases β > 1/2, β < 1/2, and β = 1/2. For practical considerations, however, only β < 1/2 is of relevance; therefore, we only consider this situation.

Now, regarding the case σ_1_ = σ_2_, it is clear from formula (9) that the optimal allocation rate is t_0_ = 1/2 because the numerator of N(t) does not depend on t, and t(1 − t) is maximized at t = 1/2. For the case σ_1_ ≠ σ_2_, we consider first 0 < σ_1_ < σ_2_. Then, it is possible to show (see Supplementary Material, Result 2) that the sample size is minimized by a t_0_ ∈ [I_1_,I_2_] with I_1_ ≤ I_2_ < 1/2. The minimizer is unique in the interval (0,1), and the bounds I_1_ and I_2_ are given by

(15) $$\;I_{1}=\frac{1}{\kappa +1},$$

(16) $$I_{2}=\frac{\sqrt{z}}{\sqrt{z}+\left(u_{1-\alpha /2}\sqrt{q}\sigma +u_{1-\beta }\sigma_{2}^{2}\right)},$$

where κ = σ_2_/σ_1_, $\sigma^{2}=\int F_{1}^{2}dF_{1}-1/4$ as in (4), q = p(1 − p), and

$$z=\left(u_{1-\alpha /2}\sqrt{q}\sigma +u_{1-\beta }\sigma_{1}^{2}\right)\left(u_{1-\alpha /2}\sqrt{q}\sigma +u_{1-\beta }\sigma_{2}^{2}\right).$$

Additionally, the following equivalence holds:

(17) $$t_{0}<\frac{1}{2}\Leftrightarrow \sigma_{1}<\sigma_{2}.$$

In the case 0 < σ_2_ < σ_1_, we obtain an analogous result for the minimizer t_0_ ∈ [I_2_,I_1_], where the bounds are the same as before. Moreover, we have a similar equivalence, namely,

(18) $$t_{0}>\frac{1}{2}\Leftrightarrow \sigma_{1}>\sigma_{2}.$$

The derivation of these two equivalences can be found in the Supplementary Material in Results 2 and 3.

From the form of the interval [I_1_,I_2_], we can see that, if κ≈1, then t_0_≈1/2. In most cases, this means that the minimum total sample size N is obtained for allocation rates close to 1/2, or the allocation rate is 1/2 because of rounding. Larger values for the type‐I error rate α or the power 1 − β lead in general to more extreme values for t_0_, ie, |1/2 − t_0_| gets larger. This can be seen from the upper bound I_2_. By increasing α or the power 1 − β, the bound I_2_ decreases (or increases for σ_1_ > σ_2_). Typically, this means that the difference |1/2 − t_0_| tends to get larger. Note that I_2_ is bounded from below (above), ie, t_0_ cannot become arbitrarily small (or large). The impact of α and β is demonstrated in simulations in Section 5.

Next, we consider the case 0 = σ_1_ < σ_2_. In the same way as before, it is possible to construct an interval for the optimal allocation rate t_0_, which is given by $\left[I_{1}^{\left(0\right)},I_{2}\right]$, where the lower bound is

(19) $$I_{1}^{\left(0\right)}=\frac{u_{1-\alpha /2}\sigma }{2u_{1-\alpha /2}\;\sigma +u_{1-\beta }\;\sigma_{2}},$$

and the upper bound is the same as in the case 0 < σ_1_. More details are given in the Supplementary Material in Result 4. An analogous result can be obtained for 0 = σ_2_ < σ_1_.

Therefore, the value of t_0_ is mainly determined by κ, which is the ratio of the standard deviations σ_1_ and σ_2_ under the alternative hypothesis. This is qualitatively different from the result of the work of Dette and O'Brien^19^ for the t‐test in a parametric location‐scale model, where the optimal allocation value is determined by the ratio of standard deviations under the null and under the alternative hypothesis. For the WMW test, the variance under null hypothesis is not really important for determining t_0_, in case of continuous distributions, eg, the variance under null hypothesis is $\sigma_{0}^{2}=1/12$.

### 3.2 Optimality of a balanced design

In the previous section, we have provided ranges for the optimal allocation proportion t_0_. There are many situations, in which balanced designs are optimal or close to optimal. In this section, we will describe classes of situations in which a balanced design minimizes the sample size. From Section 3.1, we know that

(20) $$t_{0}=\frac{1}{2}\Leftrightarrow \sigma_{1}=\sigma_{2}.$$

The right‐hand side of this equivalence can be rewritten as

(21) $$t_{0}=\frac{1}{2}\Leftrightarrow \sigma_{1}=\sigma_{2}\Leftrightarrow \int F_{1}^{2}dF_{2}=\int {\left(1-F_{2}\right)}^{2}dF_{1}.$$

Bürkner et al^1^ showed analytically that, for symmetric and continuous distributions with F_2_(x) = F_1_(x + a) and a ≠ 0, the minimal sample size is attained at t_0_ = 1/2. Such distributions satisfy the integral equation

(22) $$\int F_{1}^{2}dF_{2}=\int {\left(1-F_{2}\right)}^{2}dF_{1}.$$

However, the class of distributions satisfying Equation (22) is actually larger. Consider normalized cdfs F_1_,F_2_ for which an a∈R exists such that, for all x∈R, the following equality holds:

(23) $$F_{1}(a+x)=1-F_{2}(a-x).$$

Furthermore, let us assume 1 − β > 0.5. Then, the minimum for N(t), t ∈ (0,1) is attained at t_0_ = 1/2. This means that (23) is a sufficient but not necessary condition for t_0_ = 1/2. As an example for distributions that satisfy Equation (22) but not (23), consider F_1_ = F_2_ to be a nonsymmetric distribution.

Note that we do not assume for (23) that the distributions are stochastically ordered or symmetric. If we assume finite third moments, then Equation (23) only implies that both distributions have the same variance and their skewness has opposite signs, ie, $\nu_{F_{1}}=-\nu_{F_{2}}$, if we denote with $\nu_{F_{i}}$ the skewness of the distribution with cdf F_i_, i = 1,2.

Obviously, for a large class of distributions, the optimal allocation rate is exactly 1/2. Bürkner et al^1^ already noticed the robustness of the WMW test regarding the optimal allocation rate. When the optimal t_0_ is not equal to 1/2, it is often close to 1/2. Furthermore, the exact choice of t typically only has a small influence on the required total sample size. This applies not only to continuous and symmetric distributions but in general to arbitrary distributions.

## 4 DATA EXAMPLES

The generality of the approach proposed in this paper is demonstrated using different data examples with continuous metric, discrete metric, and ordered categorical data. In this section, we first describe the data sets. Then, the calculated sample sizes along with the actual achieved power in comparison with other sample size calculation methods are given. For all data sets, we used the prior information from one group (eg, from a previous study or from literature) to generate synthetic data for the second group based on an interpretable effect specified by a subject matter expert. For ordered categorical data, such an effect might be that a certain percentage of subjects in each category are moved to a better or worse category. For metric data, it is possible to simply use a location shift as the effect of interest. Regardless on how the effects are chosen, in the end, they all are translated into the so‐called nonparametric relative effect, which itself provides for another interpretable effect quantification, which might be useful for practitioners, in addition to, eg, a location shift effect.

For all examples, we used α = 0.05 as the type‐I error rate and provide the output from an R function, which shows the optimal t, the sample size determined for each group, and the ratio κ = σ_2_/σ_1_. Furthermore, we provide simulation results to assess the actual achieved power. The R Code is given in the Supplementary Material. For calculating the asymptotic WMW test, we used the function rank.two.samples from the R package rankFD.^31^ For all simulations performed with the statistical software R, we generated 10^4^ data sets and used 0 as our starting seed value for drawing data sets from the synthetic data. To compute the optimal allocation rate t_0_ and the sample sizes for each group, the function WMWssp_Minimize from the R package WMWssp can be used.

### 4.1 Number of seizures in an epilepsy trial

The data for the placebo group of a clinical trial published in the works of Thall and Vail^21^ and Leppik et al^20^ are shown in Table 1. As mentioned in the introduction, a relevant effect for a drug may be stated as a reduction of the number of seizures by 50%. A location‐shift model is clearly not appropriate for these data. Based on the specified relevant effect size, we can generate synthetic data. These synthetic data are generated in a way such that $F_{2}(x)={\hat{F}}_{2}(x)=F_{1}(x/q)$ for q = 0.5, ie, the empirical distribution of the generated data is equal to the alternative distribution F_1_(x/q). Hence, this leads to a nonparametric relative effect p of approximately 0.27, which is inserted into the sample size formula. For computing the sample size, it is easier to use formula (9) instead of (8). The main difference between these formulas is that we have decomposed the variance $\sigma_{N}^{*}$ into two parts, $\sigma_{1}^{*}$ and $\sigma_{2}^{*}$ (see (5)). In addition, the variance under the null hypothesis is written in terms of σ ^∗^ (see formula (4)). Then, for this sample size formula (9), we still need to calculate the variances $\sigma^{*},\sigma_{1}^{*}$, and $\sigma_{2}^{*}$. We can do that by first calculating the placements for the data according to Equation (10). Then, we use (12), (13), and (14) to obtain the quantities needed for the sample size formula.

In order to have a power of at least 80%, we need 24 subjects in each group, according to our method. When using the optimal t_0_≈0.49, we need n_1_ = 23 and n_2_ = 24 subjects. In this case, the optimal allocation only reduces the total number of subjects needed by one, in comparison with a balanced design. Applying Noether's formula in this case yields sample sizes n_1_ = n_2_ = 26. Table 2 presents results from a power simulation regarding the different sample size recommendations. Here, Noether's formula would lead to a slightly overpowered study.

**Table 2:** Power simulation for the number of seizures

Method	Sample Sizes n_1_/n_2_	Total Sample Size N	Power
Balanced	24/24	48	0.802
Unbalanced	23/24	47	0.7956
Noether	26/26	52	0.8417

### 4.2 Irritation of the nasal mucosa

In this study, two inhalable substances with different concentrations are compared with regard to the severity of the nasal mucosa damage of rats (see the work of Akritas et al^24^). The severity of irritation is described using a defect score from 0 to 3 where 0 refers to no irritation and 3 to severe irritation. For the nasal mucosa data, we have prior information for substance 1 with 2 ppm concentration. A pathologist suggests, eg, that a worsening of one score unit for 25% of the rats in categories 0, 1, and 2 is a relevant effect. This means that 25% of the rats with score 0 will be assigned score 1 and so forth. The resulting synthetic data set for substance 2 is given in Table 3. It was generated in the same way as in the previous example, ie, the empirical cdf ${\hat{F}}_{2}$ is equal to F_2_. The original data set for substance 1 has been augmented by factor 4 to obtain integer values of the samples sizes for the synthetic data for substance 2. The result of the sample size calculation is not affected by this because the relative frequencies for substance 1 remain unchanged. Then, the quantities needed for the sample size formula (9) are calculated similarly to the example form before.

**Table 3:** Number of rats with defect score 0, 1, 2, and 3

	Defect Score			
	0	1	2	3
Substance 1	64	12	4	0
Substance 2	48	25	6	1

Based on the synthetic data in Table 3, the relative effect is p = 0.599. Performing a sample size calculation with 1 − β = 0.8 and balanced groups results in sample sizes n_1_ = n_2_ = 85. For this data set, the ratio of variances κ is larger than 1; therefore, it is beneficial to assign fewer subjects to the first group (substance 1). To be more precise, the optimal allocation rate t_0_ is approximately 0.49, which leads to sample sizes n_1_ = 83 and n_2_ = 87. However, as we can see, in both cases, the total sample size is N = 170. If we apply Noether's formula,^3^ we arrive at n_1_ = n_2_ = 134, which is considerably larger than the estimated minimal sample size based on our method and leads to a remarkably overpowered study, with actual power of over 94% (see Table 4 for the simulation results). This is mainly due to ties in the data. Recall that Noether's formula was derived for continuous distributions. Our method achieves 80% power for the balanced and unbalanced design. Tang^5^ derived a sample size formula for ordered categorical data. If we use his method, we obtain that 86 rats per group are needed. The closeness of his result to ours may be taken as confirmation that our unified approach produces appropriate results also in the case of ordered categorical data.

**Table 4:** Power simulation for the nasal mucosa data

Method	Sample Sizes n_1_ **/** n_2_	Total Sample Size N	Power
Balanced	85/85	170	0.8027
Unbalanced	83/87	170	0.7999
Noether	134/134	268	0.9417
Tang	86/86	172	0.8045

### 4.3 Kidney weights

In this placebo‐controlled toxicity trial, female and male Wistar rats have been given a drug in four different dose levels. The primary outcome is the relative kidney weight in [‰], ie, the sum of the two kidney weights divided by the total body weight, and multiplied by 1000. For calculating the sample size, we consider only male rats from the placebo group and generate a suitable data set exhibiting a relevant effect for the treatment group. For generating the synthetic data of the treatment group, an expert considers a location shift of 5% of the mean from the placebo group as a relevant effect. The data are displayed in Table 5.

**Table 5:** Relative kidney weights [‰] for 16 male Wistar rats

	Relative Kidney Weight [‰]							
Placebo	6.62	6.65	5.78	5.63	6.05	6.48	5.50	5.37
Treatment	6.92	6.95	6.08	5.93	6.35	6.78	5.80	5.67

Using the data from Table 5 as our synthetic data, the nonparametric relative effect is calculated as p≈0.70. Thus, we need n_1_ = n_2_ = 30 Wistar rats to have a power of at least 80%. In this example, there is again barely any difference between using the optimal design t_0_≈0.51 (n_1_ = 31, n_2_ = 30) and a balanced allocation. Because of rounding, in this case, the optimal design even leads to a larger sample size N = 61 in comparison to N = 60 obtained using a balanced design. Noether's formula leads to sample sizes n_1_ = n_2_ = 32 in this case. The simulated power is given in Table 6. Clearly, Noether's formula again exceeds the 80% power. Our method maintains the power quite well and leads to just a slight inflation of power in the unbalanced design.

**Table 6:** Power simulation for the relative kidney weights

Method	Sample Sizes n_1_/n_2_	Total Sample Size N	Power
Balanced	30/30	60	0.7976
Unbalanced	31/30	61	0.8123
Noether	32/32	64	0.8320

### 4.4 Albumin in urine

This data set was considered by Lachin^6^ and contains albumin levels in the urine (albuminuria) of diabetic patients. The levels of albumin are rated as either normal, microalbuminuria, or macroalbuminuria. The goal of the study was to compare two treatments, with expected conditional probabilities as given in Table 7.

**Table 7:** Relative frequencies for the albumin data from the work of Lachin^6^

	Normal	Micro	Macro
Control	0.85	0.10	0.05
Experimental	0.90	0.075	0.025

For 90% power, Lachin^6^ reported a required sample size of N = 1757 (1758 because of rounding to achieve balanced sample sizes). Using our proposed method, we obtain a necessary total sample size of N = 1754 in the balanced case. For the optimal design, we obtain N = 1751 (see Table 8) with an optimal allocation rate t_0_ around 0.52. Simply using the Noether formula despite the ties, one would calculate a required sample size of N = 5334 (!), clearly leading to a much overpowered study. Based on this simulation study, the other three methods attained the nominal power. The relative effect for this data set is p = 0.474.

**Table 8:** Power simulation for the albumin in urine data

Method	Sample Sizes n_1_/n_2_	Total Sample Size N	Power
Balanced	877/877	1754	0.9054
Unbalanced	909/842	1751	0.9033
Lachin	879/879	1758	0.9029
Noether	2667/2667	5334	≈1

In the aforementioned four data examples, we have used α = 0.05 and 1 − β = 0.8 or 0.9 for the sample size calculation and power simulation according to the examples from the literature. By formula (9) and the intervals for t_0_ (Equations (15) and (16)) in Section 3.1, the choice of α and β has an influence not only on the total sample size N but also on the optimal allocation rate t_0_. In order to study the behavior of these two parameters, we have performed two simulation studies, which are described in Section 5.

## 5 SIMULATIONS FOR THE OPTIMAL DESIGN

In this section, we assess in different simulations the behavior of the optimal allocation rate *t*_0_ when changing the nominal type‐I error rate *α*, the power 1 − *β*, and the ratio of standard deviations *κ* = *σ*_2_/*σ*_1_.

**Figure 1:**
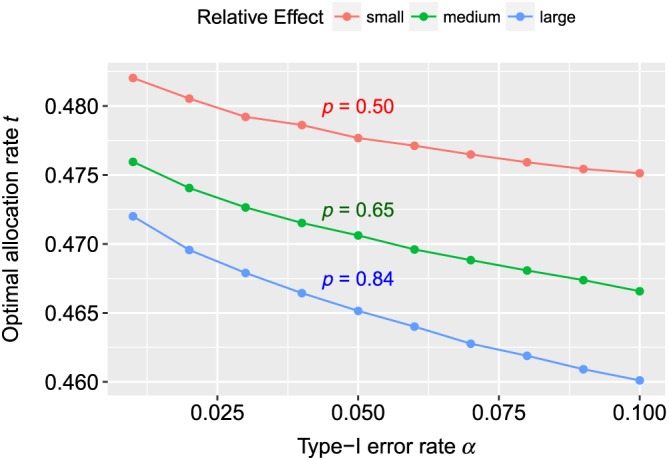
The graphic shows the values of the optimal allocation rate t_0_ for different values of type‐I error rates α where the goal is to detect a relevant effect with at least 80% power. For the reference group, we used Beta(5,5) distributions, and for the treatment group, we assumed Beta(3,i), where i = 1,2,3. The red line represents i = 3 (relative effect p≈0.5); for the green curve, we have used i = 2 ( p≈0.65), and for the red line, i = 1 ( p≈0.84) [Colour figure can be viewed at wileyonlinelibrary.com]

For simulating the influence of *α*, we used Beta(5,5) and Beta(3,*i*) distributed random numbers in the first and second group for *i* = 1,2,3. For each *α* = 0.01,0.02,…,0.1, we generated 10^6^ random numbers for each group and calculated the optimal allocation rate *t*_0_ and the total sample sizes *N*(*t*_0_) and *N*(1/2) (corresponding to a balanced design) to achieve at least 80*%* power. From the formula for the upper bound *I*_2_ of *t*_0_, we already saw (Section 3.1) that larger values for the type‐I error rate *α* would lead to a larger difference |*I*_2_ − 1/2|. While we cannot conclude from this directly that *t*_0_ will be more extreme, the optimal allocation rate will more likely tend to more extreme values, ie, the difference |*t*_0_ − 1/2| tends to become larger. We can see this behavior confirmed in Figure 1. In this simulation, we had *p*≈0.5 and *κ* = 1.35, implying *t*_0_ < 1/2 for the case *i* = 1 (red curve), *p* = 0.657 and *κ* = 1.53 (green curve), and *p* = 0.84 and *κ* = 1.98 (blue curve). Note that an effect of *p*≈0.5 makes no sense in a realistic scenario as the calculated sample size would be much too large to be of practical relevance, but we use this setting regardless just to demonstrate the behavior of *t*_0_ with regard to the effect *p*. The ratio *κ* = *σ*_2_/*σ*_1_ also has an influence on the value of *t*_0_. Hence, we chose the alternative in such a way that *κ* > 1. This means that *t*_0_ < 1/2, and if we increase *p*, then *κ* also increases. From that, we saw that more extreme effects (or larger values of *κ*) led to larger differences |*t*_0_ − 1/2|. This can also be seen from the upper bound *I*_2_.

In the data examples, we already found very little difference between using a balanced design or the optimal design. The simulation study yielded a similar observation where the maximal difference was at most 1 for the medium and large relative effect *p*, ie, max|N(t)−N(1/2)|=1. For the small effect *p*≈0.5, the maximal difference was larger but still negligible because the total sample size was very large for this setting. The detailed results are provided in the Supplementary Material.

**Figure 2:**
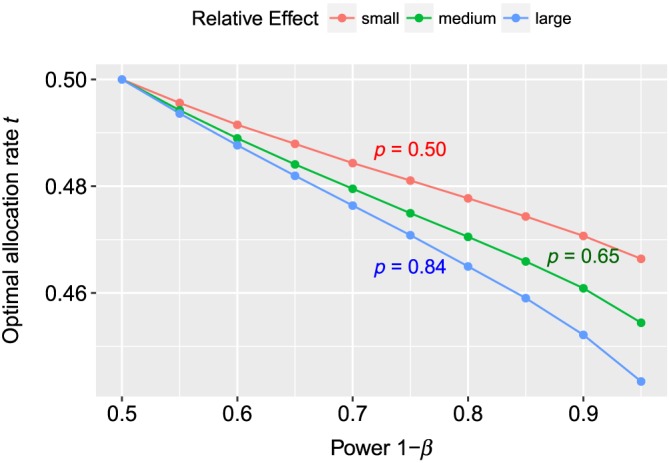
The graphic shows the values of the optimal allocation rate t_0_ for different values of the power for α = 0.05. For the reference group, we used Beta(5,5) distributions, and for the treatment group, we assumed Beta(3,i), where i = 1,2,3. The red line represents i = 3 (relative effect p≈0.5); for the green curve, we have used i = 2 ( p≈0.65), and for the red line, i = 1 ( p≈0.84) [Colour figure can be viewed at wileyonlinelibrary.com]

In a second simulation, we investigated the behavior of *t*_0_ for increasing power (or decreasing *β*). We used *α* = 0.05 and the same distributions as before. Therefore, *p* and *κ* were the same as aforementioned for the three different alternatives. As values for the power, we chose 1 − *β* = 0.5,…,0.95 and generated 10^6^ random numbers for each *β* to calculate the optimal allocation rate *t*_0_. The results are displayed in Figure 2. Obviously, for 1 − *β* = 0.5, we had *t*_0_ = 1/2 in all cases. A larger power led to more extreme values for *t*_0_, but the difference in required sample sizes between the balanced and optimal design was again negligible. The difference was again at most 1 for the medium and large relative effect *p*. Similar to the simulation from before, more extreme values of the relative effect led to larger differences |*t*_0_ − 1/2|.

## 6 DISCUSSION

In this paper, we have proposed a unified approach to sample size determination for the WMW two‐sample rank sum test. Our approach does not assume any specific type of data or a specific alternative hypothesis. In particular, data distributions may be discrete or continuous. Based on the general formula, we have also derived an optimal allocation rate to both groups, ie, to choose a value for *t* = *n*_1_/*N* such that *N* is minimized. The value of this optimal allocation rate *t*_0_ mainly depends on the ratio *κ* = *σ*_2_/*σ*_1_ (see (13) and (14) for a definition of these variances) and on *β*. The variance under the null hypothesis has no influence on *t*_0_. For *κ* > 1, we have *t*_0_ < 1/2, for *κ* < 1, we have *t*_0_ > 1/2, and for *κ* = 1, we have exactly *t*_0_ = 1/2 assuming *u*_1 − *β*_ > 0. The nominal type‐I error rate *α* only has a small impact on the value of *t*_0_. The larger *α* is, the larger is the difference |*t*_0_ − 1/2|.

We can see from the interval [*I*_1_,*I*_2_] for the optimal allocation rate *t*_0_ derived in Section 3.1 that *t*_0_ will typically be close to 1/2. This was also confirmed in some illustrative data examples in Section 4. Furthermore, the difference in required sample size between using a balanced design and using the optimal allocation design appears practically negligible.

In other words, in most cases, a balanced design can be recommended for the WMW test. In extensive simulations, we have confirmed that the new procedure actually meets the power at the calculated sample sizes quite well. In special cases, our sample size formula yields basically the same results as those by Lachin^6^ and Tang^5^ for ordinal data or Noether^3^ for continuous data (see Section 4). Matching the established results in these special cases is a desirable property for a generally valid sample size formula. However, note that, for Noether's formula, the variance under the alternative hypothesis is approximated by the variance under the null hypothesis; hence, a difference to our formula is to be expected even for continuous data (see, eg, Table 6). The advantage of our new sample size formula is that it can be used universally for different types of data. We also provide details on how to generate synthetic data based on an interpretable effect. The new procedure has been implemented in the R package WMWssp.

## Supporting information

SIM_7983‐Supp‐0001‐supp_simulation.tex

SIM_7983‐Supp‐0002‐Supplemental_R_Code_Examples.R

SIM_7983‐Supp‐0003‐Supplemental_R_Code_Figures.R

SIM_7983‐Supp‐0004‐Supplementary.pdf

SIM_7983‐Supp‐0005‐Supplementary.tex
