# Supplementary material for: Optimal sample size planning for the Wilcoxon‐Mann‐Whitney test
Source: Stat Med. 2018 Oct 8;38(3):363–75. doi: 10.1002/sim.7983 (PMC6491996; doi:10.1002/sim.7983)
Supplement: Supplementary file 4 — SIM_7983‐Supp‐0004‐Supplementary.pdf [file SIM-38-363-s004.pdf]

Supplementary Material for: Optimal Sample Size Planning for the Wilcoxon-Mann-Whitney-Test

By Martin Happ, Arne C. Bathke and Edgar Brunner

July 17, 2018

1 Derivation of the Results

1.1 Interval for the Optimal Design

Result 1. *If we assume $\sigma_1 = \sigma_2$ and $1-\beta > 0.5$ then the optimal design is given by $t_0 = \frac{1}{2}$. It is not necessary to assume $1-\beta > 0.5$ but it is convenient to do so in order to avoid a situation where $N(t) = 0$ for all $t \in (0,1)$.*

Proof. The numerator of $N(t)$ does not depend on t in this case, therefore $N(t)$ is minimized by $t_0 = \frac{1}{2}$. □

Result 2. *For $1-\beta > 0.5$ and $0 < \sigma_1 < \sigma_2$ the sample size is minimized by $t_0 \in [I_1, I_2]$ with $I_1 \leq I_2 < \frac{1}{2}$. The minimizer is unique in the interval $(0,1)$. The bounds I_1 and I_2 are given by*

$$I_1 = \frac{1}{\kappa+1}, \tag{1}$$

$$I_2 = \frac{\sqrt{z}}{\sqrt{z} + \left(u_{1-\alpha/2}\sqrt{q}\sigma + u_{1-\beta}\sigma_2^2\right)}, \tag{2}$$

with $\kappa = \sigma_2/\sigma_1$, $q = p(1-p)$ and $z = \left(u_{1-\alpha/2}\sqrt{q}\sigma + u_{1-\beta}\sigma_1^2\right)\left(u_{1-\alpha/2}\sqrt{q}\sigma + u_{1-\beta}\sigma_2^2\right)$. Additionally the following equivalence holds

$$t_0 < \tfrac{1}{2} \Longleftrightarrow \sigma_1 < \sigma_2. \tag{3}$$

Proof. First we calculate the derivative of N which is given by

$$\frac{d}{dt}N(t) = \left(u_{1-\alpha/2}\,\sigma + u_\beta\,\sqrt{\sigma_1^2\,(1-t) + \sigma_2^2\,t}\right)\frac{g(t)}{f(t)}, \tag{4}$$

where the functions f and g are defined by

$$g(t) = u_{1-\alpha/2}\,\sigma(2t-1)\sqrt{\sigma_1^2\,(1-t)+\sigma_2^2\,t} - u_\beta\,(\sigma_1^2\,(1-t)^2 - \sigma_2^2\,t^2),$$
$$f(t) = (p-\tfrac{1}{2})^2(1-t)^2t^2\sqrt{\sigma_1^2(1-t)+\sigma_2^2t^2}.$$

Only $g(t)$ has a root in $(0,1)$. Therefore, we only need to consider this function for finding the optimal t_0. To prove the equivalence we start with $t_0 < \frac{1}{2}$. In this case, $t_0 > \lambda = \frac{1}{\kappa+1}$. Because $\frac{1}{2} > t_0 > \lambda$ it follows that $\kappa > 1$. The other direction can be proved in a similar manner.

Now that we know $t_0 < \frac{1}{2}$ we can easily construct an interval for t_0. A lower bound is given by λ. For the upper bound we use the monotonic function

$$h(t) = u_{1-\alpha/2}\,\sigma(2t-1)\sqrt{q} - u_\beta\left(\sigma_1^2(1-t)^2 - \sigma_2^2t^2\right). \tag{5}$$

This function satisfies $h(t) < g(t)$ for all $t \in (0,\frac{1}{2})$ and it has exactly one root I_2 in $(0,\frac{1}{2})$. From this it immediately follows that $t_0 < I_2$.

For the uniqueness in $(0,1)$, consider a second solution $t_0' \le t_0$. It follows immediately that $t_0' > \lambda$ and consequently $\lambda \le t_0' \le t_0 \le \frac{1}{2}$. But g is strictly monotone in $(0,\frac{1}{2})$, therefore both roots are equal. □

Result 3. *For $1-\beta > 0.5$ and $\sigma_1 > \sigma_2 > 0$ the sample size is minimized by $t_0 \in [I_2, I_1]$ with $I_1 \ge I_2 > \frac{1}{2}$. The minimizer is unique in the interval $(0,1)$. The bounds are the same as in the previous theorem. Additionally the following equivalence holds*

$$t_0 > \tfrac{1}{2} \iff \sigma_1 > \sigma_2. \tag{6}$$

Proof. Similar proof as in the case $0 < \sigma_1 < \sigma_2$. □

Result 4. *For the case $\sigma_1 = 0 < \sigma_2$, we cannot apply the result from before. But using a similar idea we can find a lower bound $l(t)$ for the function $g(t)$ which is defined by*

$$l(t) = u_{1-\alpha/2}\,\sigma(2t-1)\sigma_2\,t + u_\beta\,\sigma_2^2\,t^2 \tag{7}$$

and this function only has one root in $(0,1)$, namely

$$I_1^{(0)} = \frac{u_{1-\alpha/2}\ \sigma}{2u_{1-\alpha/2}\ \sigma + u_{1-\beta}\ \sigma_2} = \frac{1}{2+\gamma}, \tag{8}$$

where $\gamma = u_{1-\beta}\ \sigma_2/\left(u_{1-\alpha/2}\ \sigma\right)$. Then an interval for the optimal design is given by $[I_1^{(0)}, I_2]$.

1.2 Optimality of a Balanced Design

From the construction of an interval for t_0 it is clear that $t_0 = 1/2$ if and only if $\sigma_1^2 = \sigma_2^2$. The equality of variances simply means

$$\int F_2^2 dF_1 - \left(\int F_2 dF_1\right)^2 = \int F_1^2 dF_2 - \left(\int F_1 dF_2\right)^2 . \tag{9}$$

From that we can easily conclude the equivalence

$$t_0 = \tfrac{1}{2} \iff \int F_1^2 dF_2 = \int (1 - F_2)^2 dF_1. \tag{10}$$

Result 5. *Let us now consider normalized cumulative distribution functions F_1, F_2 for which an $a \in \mathbb{R}$ exists such that for all $x \in \mathbb{R}$*

$$F_1(a + x) = 1 - F_2(a - x)\ . \tag{11}$$

holds, that is,

$$F_1(a + x) = 1 - F_2(a - x). \tag{12}$$

Then the optimal design is given by $t_0 = 1/2$. Furthermore if such an a exists and the expectations of the two distributions are finite, then the constant a can be explicitly calculated as

$$a = \frac{1}{2}\left(\int x dF_1(x) + \int x dF_2(x)\right), \tag{13}$$

that is, a is the average of the expected values. If the third moments are finite, then it follows from (11) that the variances of the distributions F_1 and F_2 are equal and their skewness have opposite sign. In the case $F_1 = F_2$, the assumption (11) simply means that F_1 is a symmetric distribution.

Proof. This equivalence holds since F_1 and F_2 satisfy $\int F_1^2 dF_2 = \int (1-F_2)^2 dF_1$. Equation (13) follows directly after some calculations by first considering F_1 and F_2 to be either continuous or discrete. Then (13) also holds for distributions with a continuous and discrete proportion. First we proof (13) for the discrete case. Note that from (11) we can conclude that $P(X_1 = x) = P(X_2 = 2a - x)$ holds. Then for discrete $X_1 \sim F_1$ and $X_2 \sim F_2$ the result follows from

$$\begin{aligned} EX_1 &= \sum_i x_i P(X_1 = x_i) \\ &= -\sum_i (2a - x_i) P(X_2 = 2a - x_i) + 2a \ = \ -EX_2 + 2a\ . \end{aligned}$$

The derivation for the continuous case is similar. □

2 Simulation Results

For the simulation results from Section 5 from the main manuscript, we provide here the detailed results in tables. For the first simulation we used $Beta(5,5)$ and $Beta(3,i)$ distributed random numbers in the first and second group for $i = 1,2$. For each $\alpha = 0.01, 0.02, \dots, 0.1$, we generated 10^6 random numbers for each group and calculated the optimal allocation rate t_0 and the total sample sizes $N(t_0)$ and $N(1/2)$ (corresponding to a balanced design) to achieve at least 80% power. The results for this simulation are given in Table 1.

t_0	$N(t_0)$	$N(1/2)$	p	κ	α	Effect
0.482	50344960.322	50409713.296	0.500	1.346	0.010	small
0.481	222750477.448	223086550.485	0.500	1.348	0.020	small
0.479	443308371.783	444071245.977	0.500	1.354	0.030	small
0.479	67091178.939	67213282.976	0.500	1.349	0.040	small
0.478	11583242.470	11606226.291	0.500	1.354	0.050	small
0.477	498345769.404	499384278.237	0.500	1.352	0.060	small
0.476	16344782.706	16380754.863	0.500	1.353	0.070	small
0.476	8086796.166	8105460.208	0.501	1.353	0.080	small
0.475	1089424989.407	1092041624.592	0.500	1.352	0.090	small
0.475	3308023.922	3316170.091	0.499	1.349	0.100	small
0.476	153.568	153.920	0.657	1.532	0.010	medium
0.474	131.511	131.862	0.657	1.531	0.020	medium
0.473	117.487	117.835	0.658	1.532	0.030	medium
0.472	108.820	109.170	0.658	1.532	0.040	medium
0.471	102.184	102.534	0.657	1.530	0.050	medium
0.470	96.490	96.843	0.657	1.533	0.060	medium
0.469	91.355	91.707	0.657	1.532	0.070	medium
0.468	87.552	87.906	0.657	1.532	0.080	medium
0.467	84.237	84.592	0.657	1.531	0.090	medium
0.467	80.319	80.674	0.657	1.534	0.100	medium
0.472	28.609	28.698	0.841	1.975	0.010	large
0.470	24.329	24.418	0.841	1.974	0.020	large
0.468	21.739	21.827	0.841	1.970	0.030	large
0.466	19.922	20.010	0.841	1.972	0.040	large
0.465	18.522	18.611	0.841	1.973	0.050	large
0.464	17.376	17.464	0.841	1.973	0.060	large
0.463	16.445	16.535	0.841	1.980	0.070	large
0.462	15.625	15.714	0.841	1.974	0.080	large
0.461	14.900	14.989	0.841	1.975	0.090	large
0.460	14.204	14.292	0.841	1.973	0.100	large

Table 1: Simulation results for varying type-I error probability α.

For the second simulation we investigated the behaviour of t_0 for increasing power (or decreasing β). The results are displayed in Table 2.

In both simulations we observed that an increasing power or type-I error rate leads to larger differences of $|1/2-t_0|$, that is the groups will be even more unbalanced. Similarly, from the simulations we also saw that a larger relative effect p and κ increase $|1/2-t_0|$. But the difference of total sample sizes between using a balanced design and the optimal design was negligible. For larger and medium effects this difference was at most 1, that is $|N(t_0) - N(1/2)| \leq 1$. For small effect $p \sim 0.5$ these differences are larger but they are still negligible as the total sample size itself is very large. Note that increasing p also changes κ, in our case κ increases with p.

t_0	$N(t_0)$	$N(1/2)$	p	κ	$1-\beta$	Effect
0.500	16527695.653	16527695.653	0.500	1.346	0.500	small
0.496	96422766.696	96430226.036	0.500	1.348	0.550	small
0.492	239229025.677	239297902.533	0.500	1.354	0.600	small
0.488	44000655.214	44026179.733	0.500	1.349	0.650	small
0.484	9106746.129	9115651.669	0.500	1.354	0.700	small
0.481	466636900.324	467303128.247	0.500	1.352	0.750	small
0.478	18219581.045	18255539.953	0.500	1.353	0.800	small
0.474	10804151.660	10832469.375	0.501	1.353	0.850	small
0.471	1778183697.600	1784259299.398	0.500	1.352	0.900	small
0.466	6949665.830	6980860.750	0.499	1.349	0.950	small
0.500	51.920	51.920	0.657	1.532	0.500	medium
0.494	58.363	58.371	0.657	1.531	0.550	medium
0.489	64.711	64.742	0.658	1.532	0.600	medium
0.484	72.500	72.573	0.658	1.532	0.650	medium
0.480	81.243	81.378	0.657	1.530	0.700	medium
0.475	90.959	91.185	0.657	1.533	0.750	medium
0.471	102.053	102.404	0.657	1.532	0.800	medium
0.466	116.654	117.190	0.657	1.532	0.850	medium
0.461	136.368	137.194	0.657	1.531	0.900	medium
0.454	166.142	167.507	0.657	1.534	0.950	medium
0.500	11.010	11.010	0.841	1.975	0.500	large
0.494	12.045	12.047	0.841	1.974	0.550	large
0.488	13.092	13.099	0.841	1.970	0.600	large
0.482	14.227	14.246	0.841	1.972	0.650	large
0.476	15.478	15.512	0.841	1.973	0.700	large
0.471	16.879	16.936	0.841	1.973	0.750	large
0.465	18.547	18.637	0.841	1.980	0.800	large
0.459	20.564	20.700	0.841	1.974	0.850	large
0.452	23.235	23.444	0.841	1.975	0.900	large
0.443	27.374	27.716	0.841	1.973	0.950	large

Table 2: Simulation results for varying power $1-\beta$.

3 R Code

3.1 Power Simulation

```
x1 # vector of synthetic data of first group
x2 # vector of synthetic data of second group

R <- 10^4
reject <- 0
n1 <- 299
n2 <- 299
set.seed(0)
for(i in 1:R){
z1 <- sample(x1, size = n1, prob = NULL, replace = TRUE)
z2 <- sample(x2, size = n2, prob = NULL, replace = TRUE)

df = data.frame(grp = c(rep(1,n1), rep(2,n2)), z = c(z1,z2))
df$grp <- as.factor(df$grp)

p <- rank.two.samples(z~grp, data = df, wilcoxon = "asymptotic",
info = FALSE, shift.int=FALSE,
alternative = "two.sided")$Wilcoxon$p.Value
if(p <= 0.05){
reject <- reject + 1
}
}
```

3.2 Minimize t

```
x1 # vector of synthetic data of first group
x2 # vector of synthetic data of second group
alpha = 0.05
beta=0.8
m1 <- length(x1)
m2 <- length(x2)

# ranks among union of samples:
R <- rank(c(x1,x2), ties.method="average")
R1 <- R[1:m1]
R2 <- R[m1+(1:m2)]

# ranks within samples:
R11 <- rank(x1, ties.method="average")
R22 <- rank(x2, ties.method="average")
```

```
# placements:
P1 <- R1 - R11
P2 <- R2 - R22

# effect size:
pStar <- (mean(R2)-mean(R1)) / (m1+m2) + 0.5

# variances:
sigmaStar <- sqrt(sum((R11-((m1+1)/2))^2) / m1^3)
sigma1Star <- sqrt(sum((P1-mean(P1))^2) / (m1*m2^2))
sigma2Star <- sqrt(sum((P2-mean(P2))^2) / (m1^2*m2))

sigmaStar <- sqrt(sum( (R- (m1+m2+1)/2)^2  )/(m1+m2)^3)

ss = function(t){
return((sigmaStar*qnorm(1-alpha/2) + qnorm(beta)*sqrt(t*sigma2Star^2 +
(1-t)*sigma1Star^2))^2 / (t*(1-t)*(pStar-0.5)^2))
}

# sample size with balanced groups
ss(1/2)

# optimal t
optimize(ss,interval=c(0,1), maximum=FALSE,tol = .Machine$double.eps)$minimum

# sample size given optimal t
optimize(ss,interval=c(0,1), maximum=FALSE,tol = .Machine$double.eps)$objective
```
